# The Stability of Individual Well-Being in Short Windows of Time: Women’s Perceptions across the Ovulatory Cycle

**DOI:** 10.3389/fpsyg.2017.02092

**Published:** 2017-11-30

**Authors:** Daniela Villani, Paola Iannello, Pietro Cipresso, Alessandro Antonietti

**Affiliations:** ^1^Department of Psychology, Università Cattolica del Sacro Cuore, Milan, Italy; ^2^Applied Technology for Neuro-Psychology Lab, Istituto Auxologico Italiano, Milan, Italy

**Keywords:** fertility status, ovulatory cycle, well-being, self-esteem, regulatory emotional self-efficacy

## Abstract

Empirical research on well-being has rapidly increased in recent years. One of the most dominant issue concerns the degree of cross-situational consistency and stability of well-being across time, and this is of particular relevance to women life. The aim of this study was to verify the stability of women well-being in short windows of time, specifically across menstrual cycle phases. A within-subject design with 25 normally cycling women (range: 19–26 years) was carried out. The multidimensional assessment of well-being included the administration of psychological well-being, self-esteem, and emotional self-efficacy beliefs questionnaires during both high and low-fertility phases. The results showed the stability of the level of individual well-being across menstrual cycle phases. Albeit preliminary, results indicated that women representations of their well-being do not change according to menstrual cycle. Rather, an effective organization and integration of the entire self-system appears sustained by the stability of well-being measured through a multi-componential assessment over short periods of time.

## Introduction

### The Consistency of Individual Well-Being

Empirical research in well-being intended as optimal psychological functioning (Ryan and Deci, 2001) has rapidly increased in recent years (e.g., Diener et al., 1999; Keyes et al., 2002; Seligman, 2011). Purpose, mastery, strong relationships, and self-acceptance (which are dimensions coherent with the ‘eudaimonic’ view of well-being, occurring when people’s life activities are most congruent or meshing with deeply held values and are holistically or fully engaged, e.g., Ryff, 1989; Keyes et al., 2002), as well as life satisfaction, presence of positive affect, and absence of negative affect (which are dimensions of a ‘hedonic’ view of well-being consisting of subjective happiness and concerning the experience of pleasure versus displeasure broadly construed to include all judgments about the good/bad elements of life; e.g., Diener, 2000) are most frequently used variables to assess well-being. However, evidence suggests that a positive state is more than the mere absence of negative mood or symptoms (Clark and Watson, 1991).

Even though research on well-being has traditionally focused on hedonic versus eudaimonic approach (Waterman, 1993), recent studies claimed that well-being could be best described as a multidimensional construct that includes the elements of both hedonic and eudaimonic perspectives (Keyes and Waterman, 2003; Gallagher et al., 2009; Robitschek and Keyes, 2009). In line with Karademas (2007), well-being has multiple components and positive well-being could be conceived as the cognitive and affective reactions to the perception of adequate personal characteristics and achievements, efficient interaction with the world and social integration, and positive progress in time.

Within this field of research, one of the most dominant issues concerns the degree of cross-situational consistency and stability across time that well-being possesses. Some researchers conceive well-being as a trait-like feature, which remains relatively stable across time and situations (Eid and Diener, 2004; Davern et al., 2007; Lucas and Diener, 2008), being closely linked to genetic predispositions and personality traits, in particular to extraversion, emotional stability and, to a lesser extent, consciousness (Diener and Seligman, 2002; Steel et al., 2008). On the contrary, other authors believe that well-being is a state-like characteristic, the levels of which may vary under specific life events and circumstances such as unemployment, higher income, graduation, and marriage (Reis et al., 2000; Blanchflower, 2001; Bostic and Ptacek, 2001; Lucas and Schimmack, 2009; Angner, 2010; Salinas-Jmenez et al., 2011). Nevertheless, studies focusing on the associations between malleable circumstances and well-being have mostly investigated changes in subjective well-being and have not used a multi-componential approach.

Recently, Hudson et al. (2017) showed that not only people’s global well-being (Schimmack and Oishi, 2005), as traditionally measured by self-reports, but also people’s experiential well-being, as measured by assessing lived experiences and momentary reports of well-being and including both cognitive and affective dimensions, is relatively stable over extended periods of time.

### Well-Being in Women

Gender differences literature have specifically addressed women’s well-being supporting the idea that women differ from men in the intensity of their affective experiences (Frank et al., 1991). Due to the emphasis on emotional experience within the specific social role that women have historically enacted (e.g., caretaker), they possibly have developed more appropriate skills to both recognize and express their own affect. A greater emotional sensitivity and expressiveness (Grossman and Wood, 1993; Hess et al., 2000) might justify the female (as compared to male) tendency to report more detailed representations of their own emotions (Wood et al., 1989; Barrett et al., 2000) both assessed through self-reports and objective measures (Domes et al., 2010), and higher level of happiness and well-being (Herzog et al., 1982).

Women’s well-being has been investigated in a life course perspective by focusing on the relationship between some major life events and subsequent perceived well-being. A number of studies, which were aimed at exploring the level of women’s well-being in correspondence with distinct life events (e.g., retirement: Kim and Moen, 2002; menopause: Dennerstein et al., 1994), reported a moderate stability even in the face of changing life circumstances and over long periods of time (see Diener et al., 1999). These results are consistent with the set-point theory suggesting that individuals tend to return to their baseline level of well-being even after significant life events (Headey and Wearing, 1989).

The stability of women’s well-being across short window of time has been scarcely investigated. Specifically, an interesting short period of time for monitoring women well-being is constituted by the menstrual cycle that may have important consequences on women’s psychological processes and behaviors (Neave, 2008; Durante et al., 2011, 2013; Pearson and Schipper, 2013; Iannello et al., 2015a, 2016). Among the psychological aspects that have been linked to hormonal changes during the menstrual cycle, particular emphasis has been placed on women emotional experience and self-representation. Concerning the first issue, even if some authors found changes in anxiety, depression, and responses to stress (De Ronchi et al., 2000; Clayton, 2008), these results related primarily to the pre-menstrual phase that occurs few days prior to menstruation. The current state of evidence shows little support for a specific premenstrual negative mood change occurring with any regularity in the general population (Romans et al., 2012).

Concerning self-representation, the association between menstrual cycle phases and self-esteem still appears inconsistent. Some authors found that self-esteem was lower in the premenstrual phase compared to other phases in women with premenstrual syndrome (Bloch et al., 1997; Taylor, 1999). In non-clinical populations Edmonds et al. (1995) did not find an association between self-esteem and menstrual cycle in college students, while recently Hill and Durante (2009) found that self-esteem was higher at the least fertile phase of the cycle (late luteal phase) and low around the 5 days before and 2 days after ovulation (the most fertile point of the cycle) in undergraduate students.

To date, researchers have principally focused on the implications of hormonal fluctuations across the menstrual cycle on changes in affective states and self-representation, whereas to our knowledge only one study addressed the implications for individual women’s well-being directly (Iannello et al., 2015b).

Starting from literature that sustains the stability of the whole system of well-being over long periods of time, this study aimed to verifying that the level of well-being remains stable across short window of time represented by menstrual cycle phases. We investigated well-being in high and low fertility phases through a multidimensional assessment that included: (1) psychological well-being concerning the fulfillment and self-realization of the individual in several components of positive psychological functioning; (2) self-esteem defined as the personal judgment of overall self-worth, recognized as one indicator of well-being (e.g., Baumeister et al., 2003; Mann et al., 2004); and (3) emotional self-efficacy beliefs that contribute to individuals’ psychological well-being by sustaining self-regulatory mechanisms (Bandura et al., 2003; Datu, 2012).

## Materials and Methods

### Participants

Twenty-five women among undergraduate students of the Department of Psychology at the Catholic University of the Sacred Heart of Milan were recruited. The eligibility for participation included (1) being between 20 and 30 years old, (2) having a regular menstrual cycle, (3) not being pregnant or nursing, and (4) not taking any hormonal contraceptives (e.g., pills, shots, patches, etc.). The mean age of participants was 22.4 years (*SD* = 1.9). Furthermore, we ensured that women had a level of emotional stability and extroversion consistent with normative data, as measured by the HEXACO personality inventory (Lee and Ashton, 2004), and excluded women with personality characteristics that could influence their perceived well-being (Costa and McCrae, 1980; Aghababaei and Arji, 2014). Specifically, women whose scores were above the 75th percentile on either emotional stability or extraversion were excluded from the study (*N* = 3).

### Procedure

Participants decided voluntarily to participate in the study and their anonymity was guaranteed. Each participant was asked to read and sign the consent form that confirmed their eligibility for participation and agreement to participate. Ethical compliance of the procedures was approved by the Ethical Committee at Department of Psychology of the Catholic University of Milan.

**FIGURE 1:**
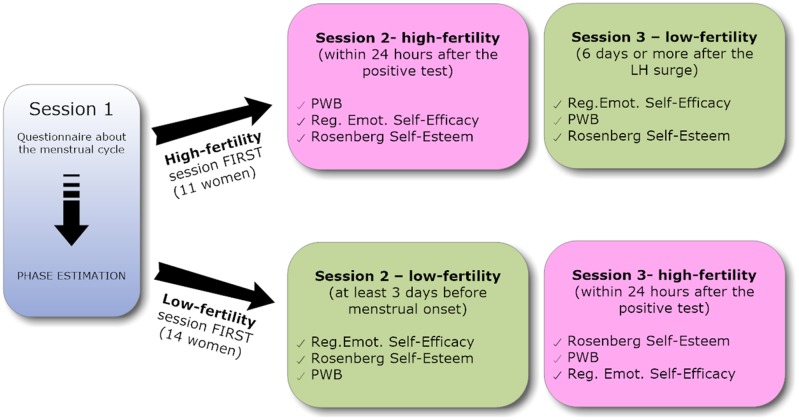
Design of the study.

Each participant was asked to complete three sessions (see **Figure 1**). In the first session, participants were asked to complete a questionnaire about their menstrual cycle to obtain information needed to estimate both the expected high-fertility and low-fertility days. Based on the information women provided about their cycle, each participant was scheduled to come into the lab for two sessions, during an expected high-fertility day and during an expected low-fertility day. To confirm the high-fertility phase, participants were instructed to use a commercially available urine-based luteinizing hormone (LH) predictor test kit at home 2 days before the expected high-fertility session and to communicate the result of the test to the researchers via email. The testing session was confirmed following a positive test, which indicated ovulation would occur within 24–36 h. After a negative test, the high-fertility lab session was re-scheduled for the subsequent ovulatory cycle. Low-fertility sessions were scheduled 6 days or more after the LH surge (if high-fertility testing session took place first) or at least 3 days before menstrual onset (if low-fertility testing session took place first). During each session, women were administered the Psychological Well-Being Scales (PWB) (Ryff and Keyes, 1995), the Regulatory Emotional Self-Efficacy Scales (Caprara and Gerbino, 2001), and the Rosenberg Self-Esteem scale (Rosenberg, 1965). In both sessions, before completing the scales, participants were specifically asked to answer the questionnaires referring to the past 3 days (*“Think about your last three days. Please, answer each item of the questionnaires referring to that short period of time”*). The order of presentation of the three scales varied randomly in each session.

### Measures

#### HEXACO Personality Inventory Revised (Lee and Ashton, 2004)

The HEXACO questionnaire comprises 100 items and incorporates the same five main personality dimensions as the well-known Big Five and Five-Factor models (Costa and McCrae, 1992), i.e., Emotionality, Extraversion, Agreeableness, Conscientiousness, and Openness to experience. Additionally, it contains the trait Honesty-Humility. Ashton et al. (2004) compared six-factor solutions observed in eight independent investigations involving seven different languages (among these the Italian language was included) and found that a similar structure emerged from each language. All items are measured on a five-point scale ranging from completely disagree to completely agree. We considered only emotionality and extraversion scales that are traditionally associated with well-being. Cronbach alphas in the present sample ranged from 0.82 (extraversion) to 0.84 (emotionality).

#### Psychological Well-Being Scale (PWB; Ryff and Keyes, 1995; Italian Version: Ruini et al., 2003)

The Psychological Well-Being Scale consists of 84 statements reflecting six distinct components of psychological well-being: individual ability to resist social pressures to think and act in certain ways and to evaluate self by personal standards (Autonomy), capacity to make effective use of surrounding opportunities and ability to choose or create contexts suitable to personal needs and values (Environmental Mastery), feeling of continue development and sense of improvement in self and behavior over time (Personal Growth), possession of warm, satisfying, trusting relationships with others (Positive Relations with Others), conviction of aims and objectives for living (Purpose in Life), acknowledge and acceptation of multiple aspects of self, including good and bad qualities (Self-Acceptance). Respondents rated statements on a scale from 1 to 7, with 1 indicating strong disagreement and 7 indicating strong agreement. In the present study, Cronbach alphas ranged from 0.70 to 0.90.

#### Rosenberg Self-Esteem Scale (Rosenberg, 1965; Italian Version: Prezza et al., 1997)

The scale comprises 10 items measuring Global Self-Worth, including both positive and negative feelings about the self. The scale is unidimensional. All items are assessed on a four-point scale ranging from strongly agree to strongly disagree. Cronbach alpha was 0.86.

#### The Regulatory Emotional Self-Efficacy Scales (Caprara and Gerbino, 2001)

The instrument assesses Self-Efficacy Beliefs in Expressing Positive Emotions (POS) and Self-Efficacy Beliefs in Managing Negative Emotions (NEG). The POS scale consists of 7 items, whereas the NEG scale includes eight items. Participants rated their self-efficacy beliefs on a five-point Likert scale from 1 (not at all well) to 5 (very well). In this study, Cronbach alphas were 0.75 (POS) and 0.72 (NEG).

### Data Analysis

We hypothesized that the level of well-being remained stable across menstrual cycle phases (low vs. high fertility). To test this hypothesis, we used the paired sample *t*-test Bayes factor (BF) to assess a ratio between the likelihood of the data given null-hypothesis and the one given the alternative one (Liang et al., 2008; Masson, 2011; Nuzzo, 2014; Rouder, 2014).

Cauchy prior width, that we used in our computation, represents the scale of the Cauchy prior density on effect size under the alternative hypothesis; the default is 0.707. On the other hand, “error %” in our results indicates the error of the Gaussian quadrature integration routine used for the computation of the BF.

Paired sample *t*-test BF is universally considered a more robust statistical test compared to *p*-value. In a recent note the American Psychological Association (2010) considered the claims of American Statistical Association and stated the importance of using also BF, among others (Wasserstein and Lazar, 2016). In this study, the choice of BF is due to the need to compare repeated measurements under the hypothesis of similarity between them. The use of BF allowed us to determine whether a model in which similarity is considered is strongly better as compared to a model where differences are considered. BF, as computed, provides the likelihood ratio of such comparison. All analyses were conducted using JASP (JASP, 2014).

## Results

First, we verified the level of emotionality and extraversion of participants. In women, both personality characteristics were consistent with normative data (emotionality: present sample *M* = 3.36, *SD* = 0.55, normative sample *M* = 3.46, *SD* = 0.50; extraversion: present sample *M* = 3.36, *SD* = 0.52, normative sample *M* = 3.25, *SD* = 0.55).

To verify that the level of well-being was stable across menstrual cycle phases, BF was calculated to provide evidence for stable individual well-being during menstrual cycle phases in comparison to the alternative model of differences in the repeated measures of well-being. The analysis showed that the data are ‘BF value’ times more likely to occur under the hypothesis that the repeated measures were equal rather than different. Psychological well-being, regulatory emotional self-efficacy, and self-esteem were compared within the two phases. As shown in **Table 1**, for example, the model that hypothesized the PWB-autonomy scale stability was more than four times better compared to the model that hypothesized a difference between the two phases.

**Table 1:** Bayesian paired samples *t*-test.

Low-fertility phase (1)	vs.	High-fertility phase (2)	BF^∗^	Error %	Effect
PWB- autonomy 1	–	PWB- autonomy 2	4.533^∗^	2.992e-4	Substantial
PWB- env mastery 1	–	PWB- env mastery 2	0.292^+^	1.802e-5	No effect
PWB- pers growth 1	–	PWB- pers growth 2	2.918^∗^	7.294e-7	Substantial
PWB- pos relations 1	–	PWB- pos relations 2	4.248^∗^	3.155e-4	Substantial
PWB- purp life 1	–	PWB- purp life 2	3.788^∗^	3.390e-4	Substantial
PWB- self-accept 1	–	PWB- self-accept 2	2.887^∗^	7.280e-7	Substantial
NEG 1	–	NEG 2	4.549^∗^	2.982e-4	Substantial
POS 1	–	POS 2	2.536^∗^	7.111e-7	Substantial
Self-esteem 1	–	Self-esteem 2	3.712^∗^	3.424e-4	Substantial

^∗^*Evidence in favor of the model of interest (similarity of measures) is considered substantial when 2.5 < BF < 10. In order to compare the relative predictive success of one model over another, the BF of the first model was divided by the BF of the other model and the value of this ratio interpreted in terms of strength of evidence using the same values as above. Practically, when the BF was substantial we can confirm that the two measures are statistically similar in contrast to the hypothesis that are different.*

*^+^BF smaller than 1 constitutes evidence in favor of the opposite model (differences of measures)*.

The results showed a substantial effect for all scales and excluded the PWB-environmental mastery that showed no effect to support null hypothesis (Jeffreys, 1961). Descriptive statistics for all scales in both low and high-fertility phase are reported in **Table 2**.

**Table 2:** Means and standard deviations of dependent variables.

Variable	Low-fertility phase		High-fertility phase	
	*M*	*SD*	*M*	*SD*
PWB- autonomy	4.33	0.54	4.34	0.64
PWB- env mastery	4.15	0.83	4.41	0.64
PWB- pers growth	4.83	0.49	4.92	0.37
PWB- pos relations	4.64	0.63	4.63	0.78
PWB- purp life	4.46	0.64	4.62	0.60
PWB- self-accept	4.06	0.78	4.22	0.88
POS	3.96	0.52	4.15	0.51
NEG	3.19	0.54	3.20	0.61
Self-esteem	3.04	0.39	3.04	0.48

## Discussion and Conclusion

The goal of the present study was to verify the stability of the level of women’s well-being across menstrual cycle phases through a within-subjects research. In line with Karademas (2007), we considered positive well-being as the cognitive and affective reactions to the perception of adequate personal characteristics and achievements, efficient interaction with the world, effective social integration, and positive progress over time. Following this view, we carried out a multidimensional assessment of well-being, including the full growth and self-realization of the individual on several components of positive psychological functioning (psychological well-being), the personal judgment of overall self-worth (self-esteem), and the self-efficacy to express positive emotions and manage negative emotions (regulatory emotional self-efficacy).

The results of our work showed that the level of individual well-being remains stable across menstrual cycle phases. Specifically, regarding psychological well-being, women’s level of well-being turned out to be consistent with normative data in relation to both gender and age (Ryff et al., 2012). Across the menstrual cycle, women reported a moderate stability in self-acceptance, positive relations with others, autonomy, purpose in life, and personal growth. Specifically, they showed moderate levels of psychological well-being related to accepting themselves and to be aware of personal limitations (self-acceptance), to be steadily connected with significant others (positive relationships), to view themselves to be living in accord with their own personal convictions (autonomy), to feel that their lives had meaning, purpose and direction (purpose in life) and to make use of their personal talents and potential (personal growth). A lack of stability was assessed in women managing of their life situations (environmental mastery) that appeared lower in low-fertility than high-fertility phase. On the one hand the global, even if moderate, stability of psychological well-being is consistent with other studies showing the evidence of mean levels of psychological well-being in adulthood (Costa et al., 1987). On the other hand, the exception of the environmental mastery respect to the other scales of the psychological well-being is consistent with recent results showing that this scale deviates from the others’ trend for example across three midlife to later-life transitions (Springer et al., 2011).

Concerning self-esteem, we found that women assessed themselves as confident and having warm feelings toward themselves and other people across the menstrual cycle. Self-esteem can be conceived as a stable cognitive and affective structure that rests upon inherited potentials and the development of which depends largely upon earlier experiences of acceptance, attachment, and recognition (Harter, 2006). Our results, which are in line with those of Edmonds et al. (1995), which suggested that self-esteem in women is not significantly influenced by hormonal fluctuations, provide a contribution to the more general debate on the stability of self-esteem, by adding evidence to the idea that self-esteem – far from being a function of specific contingencies – is generally stable over time (Trzesniewski et al., 2003).

Women’s affective self-efficacy also remained stable across the menstrual cycle. Specifically, women did not change their self-efficacy in managing negative affect and expressing positive affect. On the one hand, they assessed their individual capacity to refrain, moderate, and avoid reactions conducive to negative outcomes as stable while, on the other hand, they also assessed their capacity to voice, externalize, amplify, and share feelings conducive to sympathy, and acceptance as stable. According to Caprara et al. (2013), a robust self-esteem is associated with the firm belief that one is able to deal effectively with ones’ own affective experience. Both self-esteem and perceived affective self-regulatory efficacy have been shown to contribute significantly to the successful development and social adaptation (Bandura et al., 2003; Caprara et al., 2010; Orth et al., 2012; Balzarotti et al., 2016).

The present study has some limitations that could be overcome in further studies. First, it’s worth considering that women’s individual well-being has been assessed through self-reports. The research across the fields of psychology and other social sciences draws upon the questionnaire data on people’s perception of well-being. Thus, the findings are based on self-reported information of individuals’ beliefs about their way of thinking, feeling, and behaving. Beliefs about individuals’ psychological tendencies have formed through different experiences over time. When the respondents were asked to complete a self-report questionnaire, they referred to these consolidated and established beliefs about themselves. It could be that hormonal fluctuations in women included in this study may not have influenced their explicit set of beliefs about themselves, as measured via self-reported scales, which could account for lack of differences in the self-reported data across menstrual cycle. To overcome possible self-report biases, future research may include some additional behavioral measures, such as the individual level of stress tolerance, and utilize different data collection methods, such as the Experience Sampling Methodology (ESM), which would allow researchers to collect the longitudinal data from repeated assessments conducted in participants’ natural settings (Scollon et al., 2003). According to Hudson et al. (2017), the integration of both different types of data and different methods, and specifically of both global and experiential measures, could increase the ecological validity of the study. Alternatively, more fine-grained designs (i.e., four repeated measurements across the cycle) could allow to overcome risks of both false-positive and false-negative results (Leeners et al., 2017).

Another limitation of the current study concerns the relatively small sample size, which may limit the generalizability of this study. Even though the size of the sample was consistent with several other within-subject studies, due to the difficulty in recruiting women in specific phases of the ovulatory cycle (Derntl et al., 2008, 2013; Cantú et al., 2013; Kaighobadi and Stevens, 2013; Wu et al., 2014), we are fully aware that further larger studies are required to confirm these results.

To conclude, even if women can be susceptible to mood fluctuations due to hormonal changes, their reflections upon their experiences in various settings and the interrelated beliefs about their capabilities to manage themselves through different contexts do not appear to be influenced by the menstrual cycle. Thus, the stability we found among different self-appraisals (psychological well-being, self-esteem worth, and perceived affective self-regulatory belief) allows us to sustain an effective organization and integration of the entire self-system. On the one hand this stability appears coherent with recent studies showing that there are not consistent associations between women’s hormonal levels and cognitive processes such as attention and working memory (Leeners et al., 2017). On the other hand, these relatively stable perceptions concerning the Self may be considered as a prerequisite for women to experience and conserve a sense of coherence and self-continuity in the face of the discontinuity represented by the significant hormonal fluctuations that monthly occur.

## Author Contributions

DV conceived the study, participated in its design and coordination, performed the statistical analyses, and drafted the manuscript. PI conceived the study, participated in its design and coordination, performed the statistical analyses, and drafted the manuscript. PC participated in the design of the study and performed the statistical analyses. AA participated in the design of the study and revised the manuscript critically for important intellectual content.

## Conflict of Interest Statement

The authors declare that the research was conducted in the absence of any commercial or financial relationships that could be construed as a potential conflict of interest.
